# Clinical characteristics of peripheral blood eosinophilia in nontuberculous mycobacterial disease: a multicenter comparative retrospective study

**DOI:** 10.3389/fcimb.2026.1846229

**Published:** 2026-08-11

**Authors:** Haojun Sha, Hui Zhang, Jiehua Deng, Xianhong Chi, Tiantian Tang, Mianluan Pan, Gaoneng Fang, Shudan Tang, Wen Zeng, Jie Huang, Jianquan Zhang

**Affiliations:** 1 Department of Respiratory and Critical Medicine, The Eighth Affiliated Hospital, Sun Yat-Sen University, Shenzhen, Guangdong, China; 2 Department of Respiratory Medicine, The First Affiliated Hospital of Guangxi Medical University, Nanning, Guangxi, China; 3 Department of Respiratory Medicine, Sun Yat-Sen Memorial Hospital, Sun Yat-Sen University, Guangzhou, China; 4 Department of Tuberculosis, Nanning Fourth People’s Hospital, Nanning, Guangxi, China

**Keywords:** clinical characteristics, disseminated NTM disease, eosinophilia, nontuberculous mycobacterial disease, prognosis

## Abstract

**Background and objectives:**

Peripheral blood eosinophilia occurs in some patients with nontuberculous mycobacterial (NTM) disease, but its clinical significance remains unclear.

**Methods:**

The clinical data of 199 HIV-negative patients with NTM disease from four hospitals were retrospectively analyzed. Clinical characteristics and treatment outcomes were compared between patients with and without eosinophilia. Multivariable logistic regression and sensitivity analyses were used to evaluate the independent association between peripheral blood eosinophilia and disseminated NTM disease.

**Results:**

Of the 199 included patients, 60 had eosinophilia and 139 did not. Compared with the non-eosinophilia group, the eosinophilia group had a significantly higher proportion of disseminated disease (70.0% vs. 19.4%) and more frequently presented with systemic symptoms. Concurrent fungal infections and anti-interferon-γ autoantibody (AIGA) syndrome were more common in this group. Chest CT showed higher detection rates of solitary nodules/masses, fibrotic changes, pleural effusion, and hilar lymphadenopathy in the eosinophilia group. Baseline systemic inflammatory markers and immunoglobulin levels were also significantly elevated. After anti-infective treatment, peripheral blood eosinophil counts gradually decreased and normalized within 3 months, accompanied by the resolution of clinical symptoms and a decline in inflammatory markers. Multivariable logistic regression analysis revealed that peripheral blood eosinophilia was significantly and independently associated with disseminated NTM disease.

**Conclusion:**

NTM patients with elevated peripheral blood eosinophils are more prone to disseminated infection with augmented systemic inflammatory and immune responses, and frequently complicated with AIGA syndrome and fungal infections. Peripheral blood eosinophilia is significantly and independently associated with disseminated NTM disease in HIV-negative patients. Elevated eosinophil levels decrease after anti-NTM treatment, accompanied by marked improvements in clinical symptoms and inflammatory markers, suggesting a potential correlation between eosinophil levels and treatment response.

## Introduction

Nontuberculous mycobacteria (NTM) are opportunistic intracellular pathogens that can cause local or disseminated infections. As the global prevalence and morbidity of NTM infections continue to increase, clinicians are exhibiting heightened vigilance in their detection and management (Asayama et al., 2020; Daley et al., 2020).

Increased eosinophil counts have been noted in patients with non-tuberculous mycobacterial infections, but this observation has received little attention in previous studies. Recently, a study reported that peripheral eosinophil counts are greater in patients with NTM infection than in patients with TB. There is an association between pulmonary *M. avium-intracellulare* infections and biomarkers of Th2-type inflammation (Pfeffer et al., 2017). Eosinophils are prominent in Th2-driven immune responses, including asthma and allergic and parasitic diseases (Prakash Babu et al., 2019). As immune regulatory cells, eosinophils can recognize pathogen-associated molecular patterns (PAMPs) and damage-associated molecular patterns (DAMPs), release cytokines, regulate complement factors, interact with T and B lymphocytes, and participate in and promote both innate and adaptive immune responses (Bandeira-Melo and Weller, 2005; Rosenberg et al., 2013; Ravin and Loy, 2016; Kanda et al., 2020), thereby helping fight and eliminate specific bacterial, viral, and fungal infections (Ravin and Loy, 2016; Figueiredo and Neves, 2018; Gaur et al., 2022).

A number of studies indicate that eosinophils contribute to the host immune response against *Mycobacterium tuberculosis* (TB) (Prakash Babu et al., 2019; Bohrer et al., 2021; Bohrer et al., 2022; Zhou et al., 2024). Conversely, eosinophil-deficient mice are more susceptible to TB infection, indicating that eosinophils play a protective role in the host immune response to TB (Bohrer et al., 2021). Some humans infected with TB also experience eosinophilia, and eosinophil accumulate and infiltrate in tuberculous granulomas in their lung tissue (Bohrer et al., 2021). Furthermore, the levels of EOS-specific granule proteins including major basic protein and eosinophil-derived neurotoxin can increase in peripheral blood, which is reversed by anti-tuberculosis treatment (Moideen et al., 2018).

To date, the association between eosinophil levels and the clinical characteristics of NTM disease, along with its function in the immune response, has not been fully elucidated. The potential influence of these factors on patient prognosis remains uncertain. Hence, we performed a multicenter retrospective analysis focusing on eosinophils in NTM patients.

## Methods

### Ethical approval

This study was performed in accordance with the Helsinki Declaration of 1964 and its later amendments. This study was approved by the Ethical Review Committee of The First Affiliated Hospital of Guangxi Medical University (2024-E226-01). As this is a retrospective study, the ethics committee has approved the waiver of informed consent. All data were obtained from existing medical records and documents, involving no direct intervention or additional risks to the participants.

### Study design

#### Study population

We performed a retrospective, multicenter study at four hospitals: the Eighth Affiliated Hospital of Sun Yat-sen University, the First Affiliated Hospital of Guangxi Medical University, the Second Affiliated Hospital of Sun Yat-sen University and Nanning Fourth People’s Hospital. The clinical data of patients with NTM pulmonary disease (NTM-PD) or disseminated NTM disease admitted between May 2013 and December 2023, including age, sex, body mass index, symptom onset to diagnosis, coexisting morbidities, clinical manifestations, laboratory findings, pulmonary function, radiological features, and co-infection pathogens, were collected. The data collected at three months after treatment included symptoms, imaging findings, various blood parameters, changes in eosinophil counts over one year, and outcomes at the one-year follow-up. Radiographic features on the high-resolution computed tomography (HRCT) were evaluated by two physicians with radiographic experience, both of whom were blinded to the clinical data.

#### Definitions

The criteria for NTM-PD were as follows: NTM-PD was diagnosed according to the 2020 guidelines of the ATS/ERS/ESCMID/IDSA (Daley et al., 2020), and molecular biology methods (matrix-assisted laser desorption ionization–time-of-flight mass spectrometry, MALDI-TOF-MS; metagenomic next-generation sequencing, mNGS) were used for microbiologic evidence (Huang et al., 2019).

Criteria for disseminated NTM disease: Patients with clinical symptoms and a positive culture or molecular biology evidence for NTM from at least two sterile sites (e.g., blood, bone marrow, cerebrospinal fluid, synovial fluid, and/or extrapulmonary lymph nodes) were classified as having disseminated NTM disease (Daley et al., 2020; Sharma and Upadhyay, 2020).

The criteria for eosinophilia were as follows: eosinophilia was defined as a blood eosinophil count greater than 0.5 × 10^9^/L before and during treatment (Roufosse and Weller, 2010). We defined patients meeting the above criteria as the eosinophilic group, otherwise as the non-eosinophilic group.

Definition of follow-up endpoint outcomes: a. Cure: negative bacteriological test, no symptoms, absorption observed on radiological examination, and discontinuation of medication after clinical assessment. b. Improvement: negative bacteriological test results, improved symptoms and radiological findings, but treatment is still required after clinical assessment. c. Persistent infection: worsened or persistent symptoms and radiological findings, with or without a positive bacteriological test. d. Death: death from any cause during anti-NTM treatment (van Ingen et al., 2018; Qiu et al., 2021b).

The exclusion criteria were as follows: (1) HIV-positive patients; (2) patients with NTM colonization or contamination; (3) patients complicated with parasitic infection or other diseases that can cause eosinophil to increase due to known etiology, such as allergic diseases and Asthma; and (4) patients with a history of corticosteroid or antihistamine use in their medical records.

#### Anti-IFN-γ autoantibody assay

Serum samples were obtained under sterile conditions before the patient received antimicrobial therapy and during the active stage of the infection. Serum samples were retrieved from a serum bank and stored at -80°C. The detection of anti-IFN-γ autoantibody (AIGA) was performed using an enzyme-linked immunosorbent assay kit (Cloud-Clone Corp., Wuhan, China) whose detection range is 12–200 ng/ml. In accordance with the manufacturer’s protocols, the serum samples from patients were diluted 1:1500, and the serum samples from healthy controls were diluted 1:600 with phosphate-buffered saline. The normal range for the AIGA concentration was defined by the 99th percentile for the healthy controls and was estimated using the log-norm(al distribution. Outlying concentrations were classified as positive for AIGA (Qiu et al., 2021a).

AIGA screening was not included in routine examinations, and AIGA results were retrospectively collected as clinical data in this study. Targeted AIGA screening was only performed for NTM patients with a high clinical suspicion of AIGA syndrome at the initial diagnosis of NTM after obtaining patient consent. The enrolled patients mainly presented with disseminated, refractory, or recurrent NTM infection, accompanied by unexplained systemic inflammatory responses and typical AIGA-related complications (including fungal infections, skin lesions, osteoarticular involvement, and lymphadenopathy). They also had abnormally elevated levels of peripheral blood eosinophils, inflammatory markers, and immunoglobulins, with HIV infection and other primary immunodeficiency diseases excluded. This strict screening criterion was applied to ensure that all collected data accurately reflected the baseline clinical status of patients before treatment.

#### Specimen submission instructions

Mycobacterial culture is the foundational diagnostic gold standard and was prioritized for all suspected cases upon admission. Concurrently, baseline laboratory evaluations—including peripheral blood eosinophil differential counts—were performed at the time of culture inoculation or during the initial diagnostic workup, ensuring that these systemic parameters reflect the pre-treatment baseline clinical status (Qiu et al., 2021a).

Metagenomic next-generation sequencing (mNGS) was utilized for specimens (including sputum, bronchoalveolar lavage fluid, tissue biopsies, or sterile blood) under the following clinical indications: (1) conventional microbial cultures returned negative but clinical suspicion for NTM infection remained high; or (2) patients presented with critical illness or rapidly progressive disseminated manifestations where the protracted turnaround time of mycobacterial culture would unacceptably delay clinical management (Requena et al., 2022).

### Statistical analyses

Normally distributed values are expressed as mean ± standard deviation (SD), whereas continuous variables with skewed distributions are expressed as median and interquartile range (IQR). Categorical data are presented as raw numbers or percentages. For normally distributed data, comparisons between groups were performed using the t-test (or t-test with Welch’s correction) or ANOVA, as appropriate. For skewed data, groups were compared using the Mann-Whitney *U* test, as appropriate. Differences in categorical variables between groups were tested using the chi-square test or Fisher’s exact test. Spearman correlation coefficients were calculated between peripheral eosinophil counts and other laboratory findings in patients with NTM disease.

To avoid over-adjustment bias and collinearity within the Type 2 inflammatory pathway, clinical symptoms, radiological features, systemic inflammatory markers (C-reactive protein, albumin, globulin, and immunoglobulins G/A/M), and Type 2 immune indicators (serum IgE and CD4/CD8 ratio) were excluded from the candidate covariate pool. White blood cell and neutrophil counts were likewise excluded owing to mathematical redundancy with eosinophil counts. Among the remaining variables, those with a missing rate exceeding 30% were excluded from further analysis. For the eligible covariates, missing data were handled by multiple imputation by chained equations (MICE) using a random forest algorithm (m = 5, 10 iterations). All valid baseline covariates and the grouping variable were included in the imputation model to preserve the underlying data structure, whereas patient identifiers were excluded. Estimates derived from the imputed datasets were pooled according to Rubin’s rules.

Univariate logistic regression analysis was performed independently between independent variables and the outcome, with P-values adjusted using the Benjamini–Hochberg (FDR) method. Concurrently, 10-fold cross-validated LASSO penalized logistic regression was executed across five imputed datasets for data-driven feature reduction to screen potential key variables associated with eosinophilia, followed by multivariable logistic regression analysis to evaluate independent associations. The results of the multivariable logistic regression model were presented as adjusted odds ratios (aORs) and two-sided 95% confidence intervals (CIs). Multicollinearity diagnostics were performed using variance inflation factors (VIF), with a VIF < 5 indicating the absence of severe multicollinearity.

To evaluate the robustness of the primary findings, sensitivity analyses were performed across three dimensions: missing data handling, exposure definition, and variable selection strategy. Firstly, observations with any missing values were excluded, and the main model was re-estimated on the unimputed dataset to evaluate the sensitivity of the estimates to the multiple imputation assumption. If complete separation or extreme coefficients were detected, Firth’s penalized likelihood logistic regression was utilized to ensure stable estimation. Secondly, eosinophil count was analyzed as a continuous variable instead of a binary variable. The multivariable logistic regression model was re-fitted under identical covariate adjustments to assess whether the statistical association was altered by the choice of the cutoff value. Thirdly, variables selected by LASSO regression (with a selection frequency ≥ 50% across the five imputed datasets) were directly included to fit a logistic regression model. This approach prevented bias from complete separation and allowed for a comparison with the primary model to evaluate the potential impact of the variable selection path. For all sensitivity analyses, directional consistency and an acceptable change in the adjusted odds ratios (aORs) were utilized as the criteria for robustness.

We used SPSS (version 25.0), GraphPad Prism (version 10), and R software (version 4.4.0) for statistical analysis and graphing. Two-sided P-values < 0.05 were considered statistically significant.

## Results

### The comparisons of general characteristics of NTM patients with and without eosinophilia

We categorized patients into two groups: 60 patients with peripheral eosinophilia and 139 without. We then compared the clinical characteristics of NTM patients with and without eosinophilia, and assessed their short-term treatment efficacy as well as long-term prognosis post treatment. The detailed NTM subtypes of the two groups are shown in **Supplementary Table 1** and **2**. Among these 199 patients, the mean age was 54.9 ± 13.5 years, with a greater proportion of females (57.8%), and an overall low BMI. Patients with eosinophilia were significantly more likely to have disseminated NTM disease (70.0%) than those without eosinophilia (19.4%). The proportion of patients with NTM-PD was significantly greater in the non-eosinophilia group (80.6% vs. 30.0%). Most of the non-eosinophilia group were females (63.3%), and they were more likely to have bronchiectasis than the eosinophilia group. The eosinophilia group was more likely to have AIGA syndrome than the non-eosinophilia group. There were no significant differences in the mean age, BMI, or median time from symptom onset to diagnosis between the two groups. For all other comorbidities, the limited sample size and low event rates precluded definitive conclusions regarding between-group differences. The distributions of the rapid-growing and slow-growing subtypes were similar between the two groups (**Table 1**).

**Table 1:** Comparison of clinical characteristics between NTM patients with and without eosinophilia.

Characteristic	Eosinophilia group (n = 60)	Non-eosinophilia group (n = 139)	*P*
Gender			0.016
Male	33 (55.0)	51 (36.7)	
Female	27 (45.0)	88 (63.3)	
Age (years)	53.8 ± 10.2	55.4 ± 14.7	0.392
BMI (kg/m^2^)	20.8 ± 4.0	19.9 ± 3.2	0.122
Infection classification			
Disseminated NTM	42 (70.0)	27 (19.4)	**<.001**
NTM pulmonary disease	18(30.0)	112(80.6)	**<.001**
Classification by growth rates			0.912
RGM	20 (37.0)	50 (41.0)	
SGM	32 (59.3)	68 (55.7)	
RGM and SGM co-infection	2 (3.7)	4 (3.3)	
Bronchiectasis	11 (18.3)	86 (61.9)	**<.001**
COPD	3 (5.0)	7 (5.0)	1
Hypertension	6 (10.0)	13 (9.4)	1
Diabetes	5 (8.3)	8 (5.8)	0.717
Malignant tumors	1 (1.7)	10 (7.2)	0.178
CTD	1 (1.7)	6 (4.3)	0.677
Coronary disease	0 (0)	7 (5.0)	0.105
Hyperlipemia	0 (0)	7 (5.0)	0.105
Sweet’s syndrome	3 (5.0)	0 (0.0)	**0.026**
AIGA syndrome	27/28 (96.4)	9/14 (64.3)	**0.019**
Clinical manifestations			
Fever	28 (46.7)	41 (29.5)	**0.020**
Weight loss	19 (31.7)	23 (16.6)	**0.016**
Cough	43 (71.7)	118 (84.9)	**0.029**
Expectoration	37 (61.7)	109 (78.4)	**0.014**
Hemoptysis	9 (15.0)	49 (35.3)	**0.004**
Poor appetite	21 (35.0)	31 (22.3)	0.061
Myalgia/Arthralgia	12 (20.0)	6 (4.3)	**<.001**
Bone pain	20 (33.3)	10 (7.2)	**<.001**
Imaging bone lesion	23/25(92.0)	10/17(58.8)	**0.029**
Rash/Subcutaneous nodules	22 (36.7)	9 (6.5)	**<.001**
Superficial lymphadenopathy	31 (51.7)	10 (7.2)	**<.001**
Chest radiological features			
Solitary nodule/mass	11 (18.3)	6 (4.3)	**0.001**
Multiple nodules/masses	29 (48.3)	81 (58.3)	0.196
Bronchiectasis	11 (18.3)	86 (61.9)	**<.001**
Single cavity	7 (11.7)	14 (10.1)	0.737
Multiple cavity	1 (1.7)	20 (14.4)	**0.007**
Consolidation	54 (90.0)	121 (87.1)	0.558
Ground-glass opacities	8 (13.3)	39 (28.1)	**0.025**
Fibrotic changes	44 (73.3)	76 (54.7)	**0.014**
Pleural effusion	17 (28.3)	12 (8.6)	**<.001**
Hilar lymph node enlargement	33 (55.0)	34 (24.5)	**<.001**

Data are shown as the number (percentage) of patients or the median (interquartile range) or the average ± standard deviation. BMI, body mass index; RGM, rapidly growing mycobacteria; SGM, slowly growing mycobacteria; COPD, Chronic Obstructive Pulmonary Disease; CTD, connective tissue diseases; AIGA, Anti-interferon-γ autoantibody. Bold values indicate statistically significant results (P < 0.05).

### The comparisons of clinical manifestations of NTM patients with and without eosinophilia

More NTM patients with peripheral eosinophilia than without eosinophilia presented fever (46.7%), weight loss (31.7%), myalgia/arthralgia (20.0%), bone pain (25.0%), rash/subcutaneous nodules (36.7%), and superficial lymphadenopathy (51.7%), and some patients’ imaging suggested varying degrees of bone destruction. The non-eosinophilia group was more likely to exhibit cough (84.9%), expectoration (78.4%) and hemoptysis (35.3%). There was no significant difference between the two groups in terms of median maximum body temperature or the frequency of night sweats, chills, purulent sputum, chest pain, dyspnea, abdominal pain, poor appetite, hepatomegaly or splenomegaly. Both groups had only nonspecific digestive symptoms. The detailed results are shown in **Table 1**.

### The comparisons of chest radiological features of NTM patients with and without eosinophilia

The chest HRCT manifestations of both groups with pulmonary lesions are presented in **Table 1**. The five most common signs shown in the eosinophilia group were consolidation (90.0%), fibrotic change (73.3%), nodule/mass (66.6%), hilar lymph node enlargement (55.0%), and pleural effusion (28.3%), whereas in the non-eosinophilia group they were consolidation (87.1%), nodule/mass (62.6%), bronchiectasis (61.9%), fibrotic changes (54.7%), and ground-glass nodules (28.1%). The percentage of fibrotic changes, hilar lymph node enlargement, pleural effusion and solitary nodules/masses was significantly greater in the eosinophilia group than in the non-eosinophilia group. The non-eosinophilia group was more likely to have bronchiectasis, multiple cavities, or ground-glass opacities.

### The comparisons of laboratory findings of NTM patients with and without eosinophilia

We analyzed the data from laboratory tests before treatment (**Table 2**). The WBC, neutrophils, lymphocytes, eosinophils, platelets, globulin, immunoglobulin E (IgE), immunoglobulin G (IgG), CD4^+^ T and CD8^+^ T lymphocyte counts, procalcitonin (PCT), erythrocyte sedimentation rate (ESR) and C-reactive protein (CRP) levels were significantly higher in the eosinophilia group than in the non-eosinophilia group (P<0.05). However, variables such as the hemoglobin and albumin in the eosinophilia group were lower than the normal reference values, and lower than those in the non-eosinophilia group. We collected pulmonary function test data from 49 patients, including total lung capacity (TLC% predicted), forced expiratory volume in one second (FEV_1%_ predicted), ratio of forced expiratory volume in one second to forced vital capacity (FEV_1_/FVC %), the diffusing capacity for carbon monoxide per liter of alveolar volume (DLCO/VA% predicted), and no statistically significant differences were shown in these indicators between the two groups.

**Table 2:** Comparison of laboratory findings between NTM patients with and without eosinophilia.

Variable	Eosinophilia group (n = 60)	Non-eosinophilia group (n = 139)	P value
WBC (×109/L)	13.0 (8.2, 18.3)	6.7 (5.0, 8.6)	**<.001**
N (×109/L)	8.7 (4.9, 13.7)	4.2 (3.1, 6.0)	**<.001**
L (×109/L)	1.6 (1.3, 2.3)	1.4 (1.1, 1.9)	**0.014**
EOS (×109/L)	0.8 (0.6, 1.16)	0.1 (0.1, 0.2)	**<.001**
PLT (×109/L)	367.0 (262.5, 515.3)	235.0 (185.0, 301.0)	**<.001**
HGB (g/L)	103.1 (76.1, 120.0)	120.3 (108.0, 129.0)	**<.001**
ALB (g/L)	31.15 (27.9, 37.7)	37.48 (34.0, 41.3)	**<.001**
GLO (g/L)	36.33 (30.5, 43.5)	31.20 (27.2, 35.4)	**0.001**
Immunoglobulin	N=37	N=61	
IgG (g/L)	16.7 ± 6.2	14.2 ± 5.6	**0.043**
IgA (g/L)	2.9 ± 1.4	2.4 ± 1.0	0.117
IgM (g/L)	0.99 (0.7, 1.5)	1.02 (0.7, 1.5)	0.921
IgE (IU/ml)	146.6a (45.6, 291.4)	27.32b (13.4, 68.0)	**<.001**
CD4^+^ T (/μL)	757c (542.3, 1057.5)	485d (360.0, 778.0)	**0.001**
CD8^+^ T (/μL)	532c (386.3, 709.0)	357d (214.0, 499.0)	**0.001**
PCT (ng/mL)	0.13 (0.09, 0.43)	0.05 (0.03, 0.10)	**<.001**
ESR (mm/h)	74.5 (40.0, 94.8)	30.5 (11.8, 56.3)	**<.001**
CRP (mg/L)	54.93 (10.5, 109.2)	8.05 (2.9, 27.8)	**<.001**
BDG (pg/mL)	10 (10.0, 11.8)	10 (10.0, 20.7)	0.872
GM test (pg/mL)	0.32e (0.2, 0.5)	0.32f (0.2, 0.7)	0.903
Pulmonary function test	N=13	N=36	
TLC (% predicted)	85.4 (79.3, 89.5)	85.06 (80.4, 97.7)	0.960
FEV1 (% predicted)	96.6 ± 15.5	91.5 ± 23.7	0.477
FEV1/FVC (%)	79.5 ± 6.1	76.6 ± 10.5	0.352
DLCO (% predicted)	82.9 (70.3-95.2)	87.8 (75.8-93.6)	0.465

^a^n=33, ^b^n=56, ^c^n=40, ^d^n=44, ^e^n=55, ^f^n=57. Data are shown as the number (percentage) of patients or the median (interquartile range) or the average ± standard deviation.

Definition of abbreviations: WBC, white blood cell; N, neutrophil; L, lymphocyte; EOS, eosinophil;PLT, platelet; HGB, hemoglobin; ALB, albumin; GLO, globulin; IgG, Immunoglobulin G;IgA, Immunoglobulin A; IgM, Immunoglobulin M; IgE, Immunoglobulin E; PCT, procalcitonin; ESR, erythrocyte sedimentation rate; CRP, C-reactive protein; BDG, 1, 3-β-D-glucan assay; GM test, galactomannan test; TLC,total lung capacity; FEV_1_, forced expiratory volume in one second; FVC, forced vital capacity; DLCO/VA, diffusing capacity for carbon monoxide per liter of alveolar volume. Bold values indicate statistically significant results (P < 0.05).

### The increased frequency of fungal co-infection in NTM disease patients with eosinophilia

**Figure 1:**
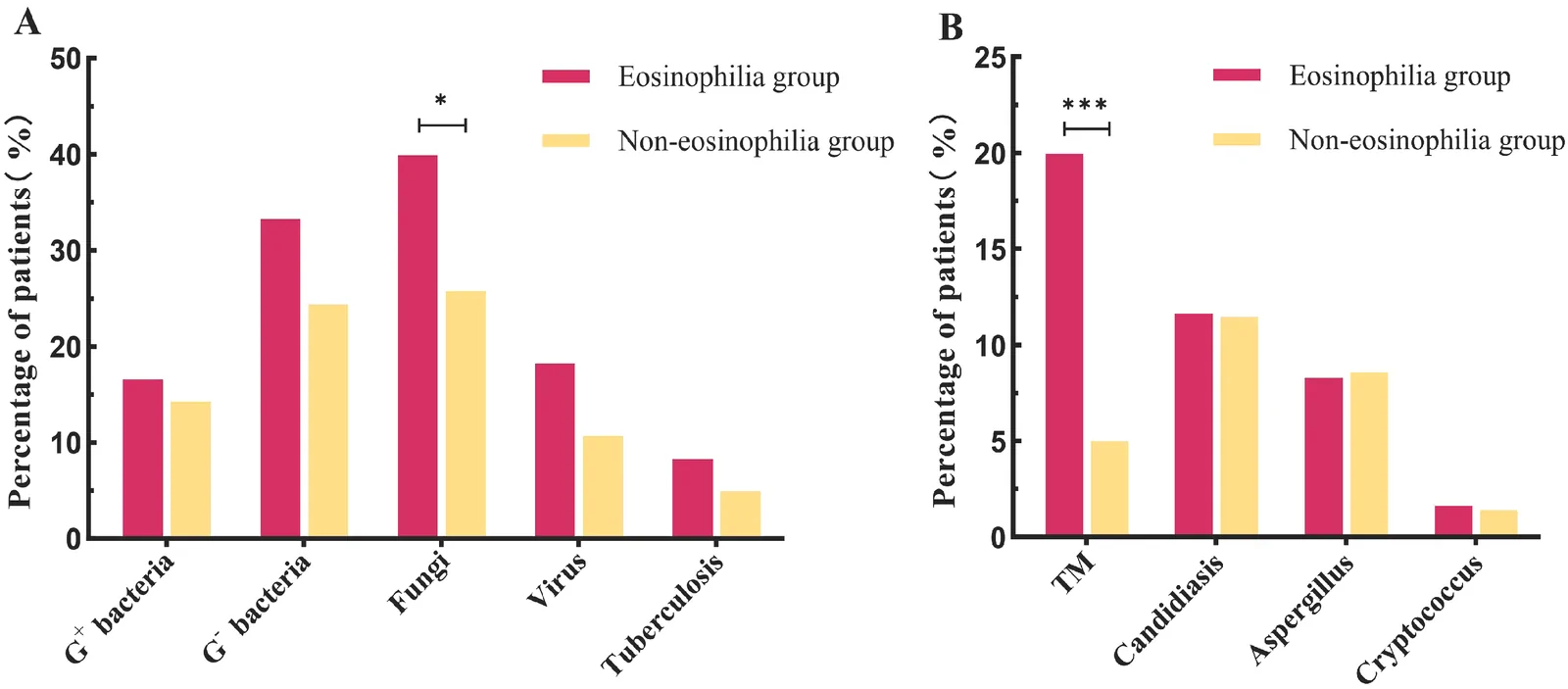
The frequency of co-infection pathogens in NTM disease patients with eosinophilia. **(A)** The co-infection pathogens in eosinophilia group and non-eosinophilia group. **(B)** The specific subtypes of fungal infections in eosinophilia group and non-eosinophilia group. *p < 0.05, ***p < 0.001. Each patient may have more than one subtype infection.

Many patients with NTM disease experience co-infections with other pathogens at different stages of the disease. The most common co-infection type was fungal infection (35.0%), followed by Gram-negative (G^-^) bacteria (27.0%), Gram-positive (G^+^) bacteria (15.5%), viruses (13.0%), and tuberculosis (6.0%) (**Figure 1A**). We found that NTM patients with eosinophilia were more likely to have fungal infections (40.0% vs. 25.9%), especially infections caused by *Talaromyces marneffei* (20.0% vs. 5.0%) (P < 0.001, **Figure 1B**, **Supplementary Table 3**).

### The positive correlation between eosinophil count and AIGA titer in NTM disease patients

**Figure 2:**
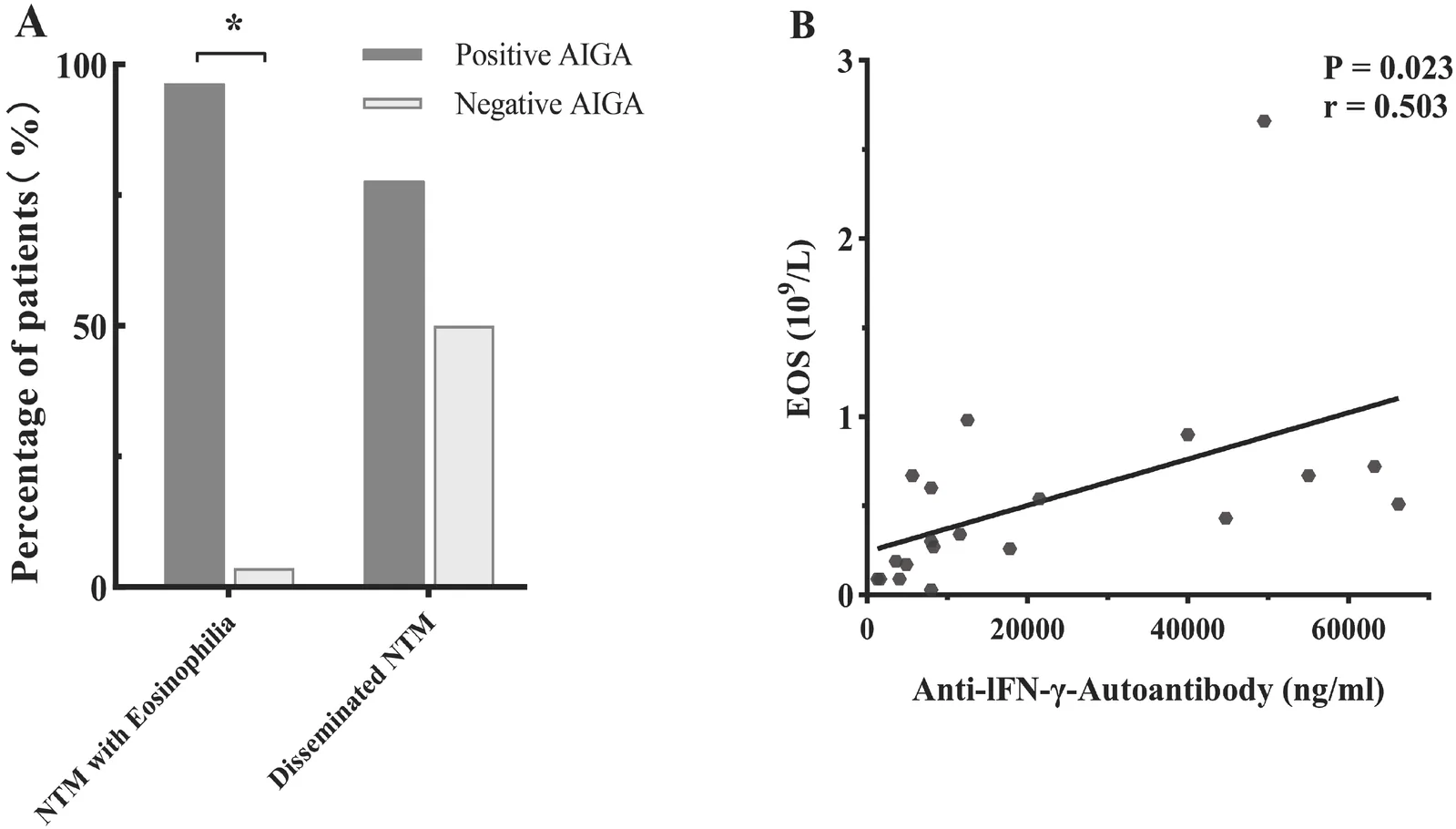
The correlation between eosinophil count and AIGA titer in NTM disease patients. **(A)** Comparison of AIGA-positive and AIGA-negative NTM patients with eosinophilia and disseminated NTM. **(B)** The Spearman correlation analysis between AIGA titer and the count of eosinophil. *p<0.05.

In the baseline comparison of the entire cohort, the eosinophilia group had a significantly higher prevalence of AIGA syndrome (96.4% vs. 64.3%, P = 0.019; **Table 1**). Based on these findings, we further screened 20 patients with available quantitative AIGA titer data from 42 enrolled patients for correlation analysis. The analysis revealed that the proportion of patients with peripheral blood eosinophilia was higher in the AIGA-positive group than in the AIGA-negative group (**Figure 2A**). The difference in the proportion of concurrent disseminated NTM between the two groups did not reach statistical significance (**Figure 2A**), which may be attributable to insufficient statistical power given the small sample size of the negative control group (n = 6). Spearman rank correlation analysis was further performed to assess the quantitative relationship between the absolute peripheral blood eosinophil count and the AIGA titer. The analysis revealed a statistically significant, moderate positive correlation (r = 0.503, P < 0.05, **Figure 2B**). The modest strength of this correlation suggests that although higher AIGA levels are accompanied by higher eosinophil levels, this autoantibody may represent only one of the multiple factors involved in the systemic immune profile, rather than the sole determinant of peripheral eosinophil expansion in NTM disease.

### The clinical outcomes of NTM patients and dynamic changes in eosinophil counts after treatment

**Figure 3:**
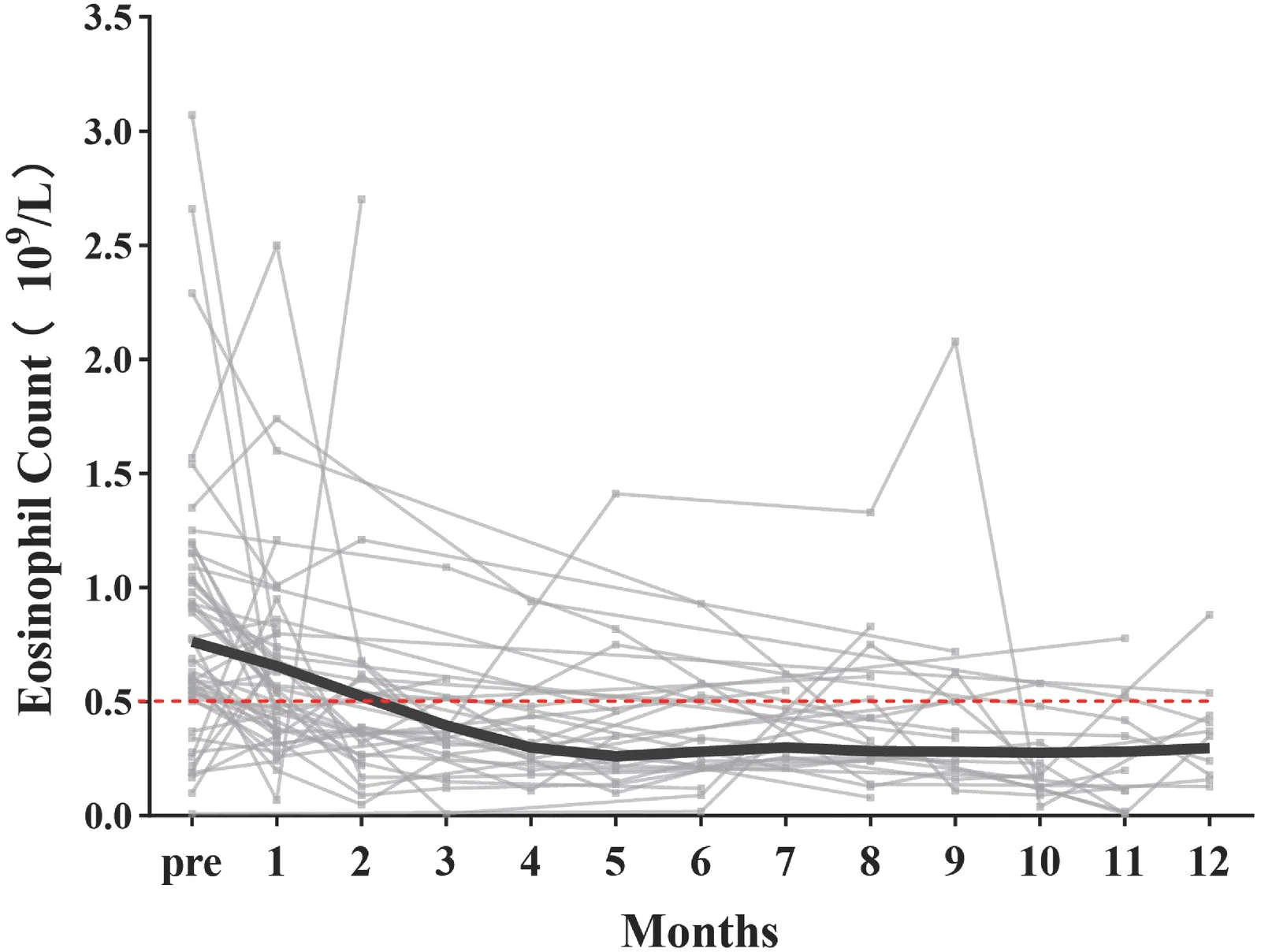
The dynamic changes in eosinophil counts in NTM patients with eosinophilia after treatment.

Monthly peripheral eosinophil counts from 50 patients with eosinophilia were analyzed over a one-year treatment period to track their dynamic changes (**Figure 3**). Notably, we observed a continuous decline in the overall eosinophil count as treatment progressed, with the counts returning to normal levels by approximately three months.

The gray lines represent data for each individual patient. The black line is a smoothed curve created using the median eosinophil count at each time point. Pre: Before treatment; 1-12: 1–12 months after treatment.

**Figure 4:**
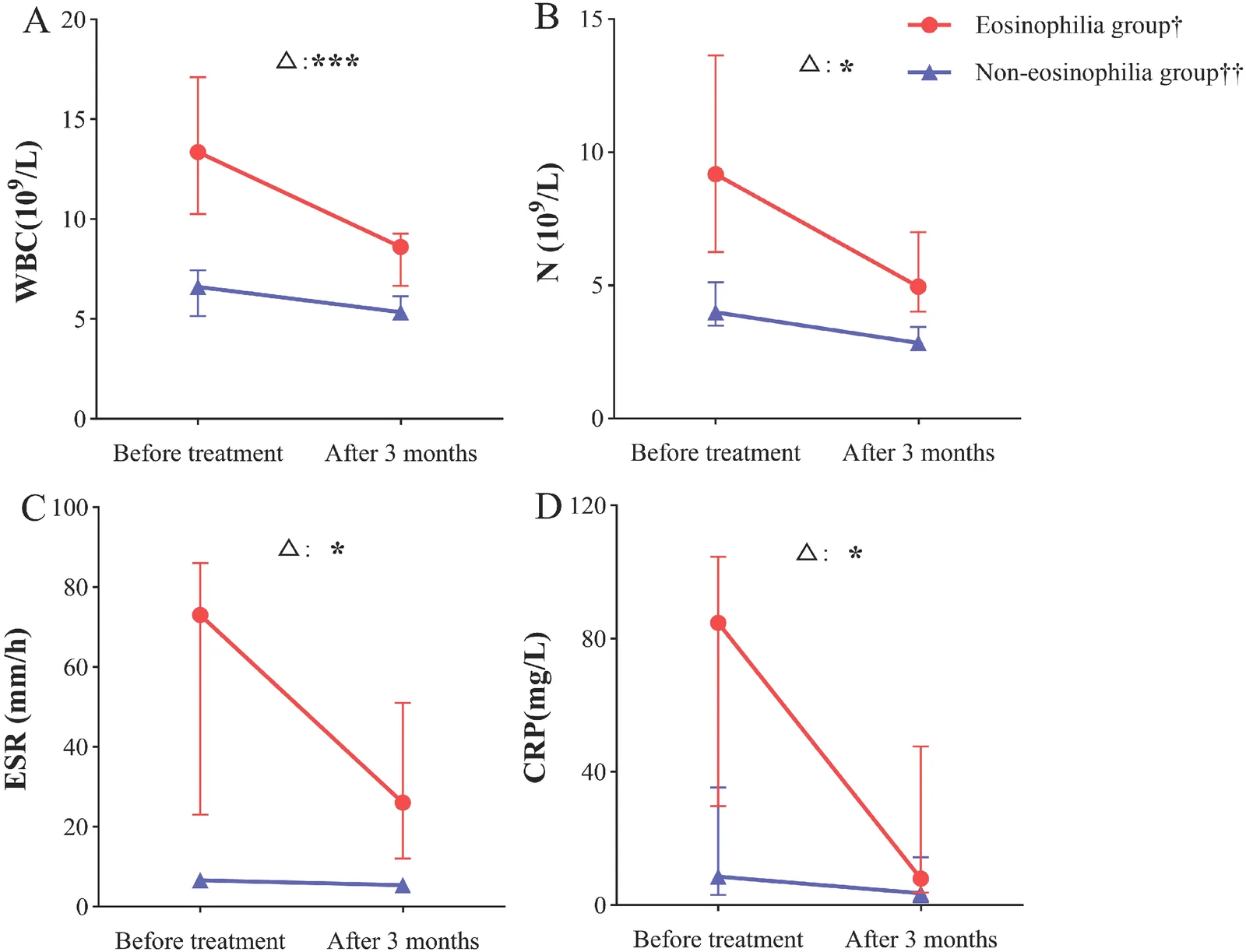
Changes in inflammatory parameters before and after treatment in eosinophilia group and non-eosinophilia group. The comparisons of **(A)** WBC, **(B)** neutrophils (N), **(C)** ESR, and **(D)** CRP before and after treatment for three months in the two groups. △ represents the difference in index values before and after treatment. ^†^N = 33, ^††^N = 52. *p<0.05, ***p<0.001.

After three months of treatment, the symptom improvement rate was significantly greater in the eosinophilia group (**Table 3**). A greater proportion of patients in the non-eosinophilia group showed no change in symptoms or radiological findings. When we analyzed laboratory indicators after three months of treatment, the eosinophilia group showed greater reductions (△) in WBC, neutrophils, ESR, and CRP level (**Figure 4**). There were no differences in the improvements in lymphocytes, hemoglobin, IgE, CD4^+^T cell, CD8^+^T cell, or albumin levels between the two groups (**Supplementary Figure 1**).

**Table 3:** Changes in symptoms and chest imaging in two groups after three months of treatment.

Variables	Eosinophilia group	Non-eosinophilia group	P
After 3 months of treatment			
Symptoms	N=45	N=82	
Improve	40 (88.9)	60 (73.2)	0.038
Unchange	0 (0.0)	12 (14.6)	0.017
Worsen	5 (11.1)	10 (12.2)	0.856
Chest imaging	N=42	N=77	
Improve	29 (69.1)	44 (57.1)	0.203
Unchange	4 (9.5)	20 (26.0)	**0.033**
Worsen	9 (21.4)	13 (16.9)	0.542
After 1 year of treatment			
Prognosis and outcomes	N=47	N=81	
Cured	7 (14.9)	6 (7.4)	0.227
Improved	36 (76.6)	53 (65.4)	0.188
Persistent or relapse infection	4 (8.5)	20 (24.7)	0.032
Death	0 (0.0)	2 (2.5)	0.528

Bold values indicate statistically significant results (P < 0.05).

We collected the outcomes of 128 patients at the one-year follow-up, including cure, improvement, persistent infection, and death. Two patients died, both of whom were in the non-eosinophilia group. As shown in **Table 3**, after 3 months of treatment, a greater proportion of patients in the eosinophilia group than in the non-eosinophilia group were Symptoms improved (P = 0.038), in contrast, the proportion of patients with unchanged symptoms (P = 0.017) and chest imaging (0.033) was significantly higher in the non-eosinophilia group than in the eosinophilia group. After 1 year of treatment, the eosinophilia group had a significantly lower rate of persistent infection or recurrence (8.5%) than the non-eosinophilia group (24.7%, P = 0.032).

### Independent association between disseminated NTM disease and peripheral blood eosinophilia

A total of 199 patients with NTM were included in our study. Patients were categorized into disseminated NTM and NTM pulmonary disease groups, and we compared their demographic characteristics, clinical presentations, chest imaging findings, laboratory findings, and co-infection status (**Supplementary Table 4**).

To evaluate the independent statistical association between disseminated NTM disease and peripheral blood eosinophilia, a multivariable logistic regression model was constructed including clinical covariates selected based on prior and aforementioned knowledge and statistical criteria. After adjusting for potential confounders, peripheral blood eosinophilia was significantly and independently associated with disseminated NTM disease (aOR = 5.61, 95% CI: 2.44–12.88, P < 0.001). Concurrently, fungal co-infection was also positively associated with the disease status (aOR = 4.45, 95% CI: 1.99–9.96, P < 0.001), whereas bronchiectasis exhibited a negative statistical association (aOR = 0.17, 95% CI: 0.07–0.41, P < 0.001). The remaining adjusted covariates showed no statistically significant associations: age (aOR = 0.99, 95% CI: 0.96–1.02, P = 0.384), sex (aOR = 0.98, 95% CI: 0.44–2.19, P = 0.953), connective tissue diseases (aOR = 3.83, 95% CI: 0.56–26.24, P = 0.171), viral co-infection (aOR = 2.20, 95% CI: 0.73–6.60, P = 0.160), and COPD (aOR = 2.92 (according to the **Table 4**, aOR of COPD), 95% CI: 0.12–4.89, P = 0.780). More detail can be seen in **Table 4**.

**Table 4:** Multivariate analysis of the risk factors for disseminated NTM disease.

Characteristic	aOR	95%CI	P
Eosinophilia	5.606	2.440-12.881	**< 0.001**
Age	0.987	0.959-1.016	0.383
Female	0.976	0.436-2.185	0.952
Bronchiectasis	0.171	0.072-0.406	**< 0.001**
COPD	2.924	0.121-4.891	0.780
CTD	3.825	0.558-26.237	0.170
Virus co-infection	2.195	0.730-6.601	0.160
Fungi co-infection	4.448	1.987-9.961	**< 0.001**

COPD, Chronic Obstructive Pulmonary Disease; CTD, Connective Tissue Diseases; aOR, adjusted odd ratio.

Bold values indicate statistically significant results (P < 0.05).

### Sensitivity analyses

To evaluate the robustness of the primary findings, sensitivity analyses were performed across three pre-specified dimensions. First, in the complete-case analysis excluding all observations with missing values, peripheral blood eosinophilia remained significantly associated with disseminated NTM disease (aOR = 4.95, 95% CI: 2.12–11.93, P < 0.001), with bronchiectasis and fungal co-infection showing consistent statistical correlations (**Supplementary Table 5**). Second, when evaluating the exposure as a continuous variable, higher eosinophil counts were consistently associated with disseminated NTM disease (aOR = 3.14, 95% CI: 1.49–7.59, P = 0.006) (**Supplementary Table 6**). Third, in the sensitivity analysis testing the alternative LASSO-driven variable selection strategy, the independent association for peripheral blood eosinophilia persisted (aOR = 4.69, 95% CI: 2.05–11.04, P < 0.001), while bronchiectasis, fungal co-infection, hypertension, and viral co-infection also demonstrated significant statistical associations. Across all three dimensions, both the direction and magnitude of the estimated association for eosinophilia remained stable (**Supplementary Table 7**).

## Discussion

The NTM cohort enrolled in this study is reasonably representative. The results showed that NTM-PD occurred predominantly in middle-aged women, with bronchiectasis being the most common comorbidity. The nodular bronchiectatic form accounted for the vast majority of imaging findings. Additionally, 34.0% of patients had a concurrent fungal infection, with *Talaromyces marneffei* being the most common pathogen. A single-center study with a 7-year follow-up reported that advanced age, bronchiectasis, COPD, and bronchiectasis with a nodular imaging pattern were the main clinical features of NTM-PD (Liu et al., 2023). A large-sample study from the United States also showed that NTM infection was most prevalent in individuals over 50 years of age, with cardiovascular disease as the most common underlying condition, followed by bronchiectasis and COPD (Billinger et al., 2009). Based on these general characteristics, this study further performed a stratified analysis by peripheral blood eosinophil levels. We found that in the eosinophilia group, the proportion of disseminated NTM was significantly higher. A subset of patients had concomitant AIGA syndrome, the proportion of which even exceeded that of bronchiectasis. Moreover, both the symptom improvement rate and the clinical cure rate after treatment were higher. Finally, after adjusting for key confounding variables in the multivariable logistic regression model, peripheral blood eosinophilia remained significantly and independently associated with disseminated NTM.

Patients with eosinophilia presented more prominent clinical manifestations, such as high fever, weight loss, myalgia/arthralgia, bone pain, skin lesions, and superficial lymph node enlargement. Radiological findings reveal bone destruction, and chest imaging often showed solitary nodules or masses, fibrotic changes, pleural effusion, and hilar lymph node enlargement. These patients often present with symptoms of systemic infection and extrapulmonary damage, as well as nonstructural lung damage and extrapulmonary invasion. In the non-eosinophilia group, patients mainly exhibited respiratory symptoms such as cough, expectoration, and hemoptysis. Chest CT typically showed structural damage, such as bronchiectasis, multiple cavities, and ground-glass opacities. One reason for these differences might be that patients with normal eosinophil counts are more likely to have NTM lung disease, while those with eosinophilia are more likely to have disseminated NTM disease, manifesting as multiple local invasions, systemic infections, and consumptive symptoms. Therefore, when NTM infection is accompanied by eosinophilia and related clinical symptoms, the possibility of disseminated NTM infection should be considered. Eosinophilia can cause skin issues and swollen lymph nodes (Requena et al., 2022). Hence, it’s important to perform skin and lymph node biopsies and other tests to determine if these symptoms are due to non-tuberculous mycobacterial infection or eosinophil infiltration.

In the eosinophilia group, AIGA syndrome was the most common comorbidity. This may be associated with the finding that over half of the patients in this group had disseminated NTM disease. Accumulating evidence indicates that AIGA syndrome is a core risk factor for disseminated NTM disease in Asian HIV-negative patients (Browne et al., 2012; Chi et al., 2016; Aoki et al., 2018; Hong et al., 2020). Our previous study also confirmed that NTM infection is most prevalent among AIGA-positive individuals (Qiu et al., 2022). These anti-cytokine autoantibodies neutralize IFN-γ, thereby impairing macrophage lysosomal function and weakening host defense against mycobacteria. Furthermore, this process may induce Th1 deficiency, Th1/Th2 imbalance, and a shift toward Th2 responses, ultimately leading to aberrant IL-5 secretion that drives eosinophil proliferation and activation (Jasenosky et al., 2015; Lyadova and Panteleev, 2015; Eberl, 2016). Fungal co-infection, especially *Talaromyces marneffei* infection, was more common in the eosinophilia group. Host clearance of intracellular fungi such as *Talaromyces marneffei* also critically depends on the IFN-γ axis. The presence of AIGA renders the host equally susceptible to NTM and *Talaromyces marneffei*, providing the pathophysiological basis for dual co-infection (Zhang et al., 2023). Concurrently, extracellular proteases secreted by co-infecting *Aspergillus* or *Talaromyces marneffei* act as Type 2 innate immune activators. They directly promote Th2 responses independently of adaptive immunity, synergistically elevating and sustaining high peripheral eosinophil levels (Lilly et al., 2014; Knight et al., 2020). The specific mechanisms underlying eosinophil elevation in the context of NTM and fungal co-infection remain to be elucidated.

Eosinophil rapidly accumulates in the lungs to form granulomas during human or animal infections with TB (Bohrer et al., 2021). Although we did not find eosinophilic granulomas in NTM patients, we did find increased infection and inflammatory marker levels in the eosinophilia group, indicating systemic inflammation. Additionally, our study revealed increased CD4+ and CD8+ T-cell counts in the eosinophilia group. Eosinophils can express major histocompatibility complex class II (MHC-II) and function as antigen-presenting cells (APCs) to process antigens, stimulating T cells by producing cytokines related to T-cell polarization (Bandeira-Melo and Weller, 2005; Ravin and Loy, 2016). Eosinophil-derived interleukin(IL)-4 promotes the generation of CD8+ T-cell memory, which contributes to potentiate antibacterial immunity (Zhou et al., 2024). We speculate that eosinophils promote CD4+ and CD8+ T cells, as well as an enhanced immune response in NTM infections, and participate in the defense against pathogens. The specific mechanisms remain to be further investigated.

Following anti-NTM therapy, peripheral blood eosinophil counts in patients with eosinophilia normalized. At the three-month landmark of treatment, the eosinophilia group exhibited a higher symptom improvement rate and more pronounced reductions in systemic inflammatory markers. The normalization of eosinophil counts during treatment may reflect an effective host immune clearance response. However, it should be noted that the higher prevalence of irreversible structural damage, such as bronchiectasis and cavities, in the non-eosinophilia group may be an important reason for the poorer treatment response observed in that group. Meanwhile, clinical treatment bias cannot be ruled out, because the eosinophilia group had a higher proportion of disseminated cases, and these patients may have received more aggressive or prolonged anti-NTM therapy, thereby partially amplifying the therapeutic advantage observed in this group. Therefore, the prognostic value suggested by eosinophilia cannot currently be considered independent of the confounding effects of baseline pulmonary lesions and differences in treatment intensity.

The core of multivariable logistic regression analysis lies in controlling for key confounding variables to verify whether the statistical association between peripheral blood eosinophilia and disseminated NTM disease exists independently. Our findings demonstrate that after adjusting for the effects of traditional predisposing features such as bronchiectasis, this association remains statistically significant and highly robust. This indicates that peripheral blood eosinophilia cannot be fully explained by known factors such as airway structural defects, but rather represents an independent, systemic phenotypic feature. Consequently, these statistical findings further confirm that peripheral blood eosinophilia has a significant independent association with disseminated NTM disease.

This study has several limitations. First, due to its retrospective design, potential selection bias and missing clinical data may affect the accuracy of the descriptive and multivariable analyses, although sensitivity analyses were performed to confirm the robustness of the primary findings. Second, as an observational study, the identified association between peripheral blood eosinophilia and disseminated NTM disease cannot establish direct causality. Third, eosinophil differential counts in sputum or bronchoalveolar lavage fluid (BALF) were unavailable; therefore, the relationship between the local airway eosinophilic microenvironment and the clinical outcomes of NTM disease could not be determined. Fourth, the multicenter cohort was drawn exclusively from South China, an endemic area for *Talaromyces marneffei* infection. This specific regional immunological background may limit the generalizability of our findings to populations lacking these epidemiological features. Fifth, the follow-up period for evaluating treatment response was limited to three months, which is insufficient to determine the long-term prognosis or recurrence patterns of this chronic disease. Sixth, due to the limited sample size, the multivariable logistic regression model only incorporated some known confounders, failing to fully control for all potential confounders. Seventh, despite strict inclusion and exclusion criteria, control for confounding, and sensitivity analyses, the limitation of high case heterogeneity could not be overcome. Nevertheless, this is the first large-sample multicenter study to analyze the clinical and immunological characteristics of the eosinophilic phenotype in patients with NTM infection. This study innovatively explores the clinical features and prognosis of NTM patients with eosinophilia. The findings lay a foundation for further exploring the specific mechanisms and signaling pathways of eosinophils involved in NTM infection.

## Conclusion

NTM patients with elevated peripheral blood eosinophils are more prone to disseminated infection with augmented systemic inflammatory and immune responses, and frequently complicated with AIGA syndrome and fungal infections. Peripheral blood eosinophilia is significantly and independently associated with disseminated NTM disease in HIV-negative patients. Elevated eosinophil levels decrease after anti-NTM treatment, accompanied by marked improvements in clinical symptoms and inflammatory markers, suggesting a potential correlation between eosinophil levels and treatment response. In contrast, NTM patients without eosinophilia more commonly present with respiratory symptoms, frequently complicated by bronchiectasis and more severe pulmonary structural damage.

## Data Availability

The original contributions presented in the study are included in the article/**Supplementary Material**. Further inquiries can be directed to the corresponding author.

## Supplementary material

Supplementary Figure 1 Changes in lymphocyte (L), albumin (ALB), hemoglobin (HGB), CD4^+^ T, and CD8^+^ T lymphocyte counts and immunoglobulin E (IgE) levels before and after treatment in eosinophilia group and non-eosinophilia group. ^†^N = 33, ^††^N = 52. No statistical difference was found.
