# Supplementary material for: Clinical characteristics of peripheral blood eosinophilia in nontuberculous mycobacterial disease: a multicenter comparative retrospective study
Source: Front Cell Infect Microbiol. 2026 Aug 11;16:1846229. doi: 10.3389/fcimb.2026.1846229 (PMC13503238; doi:10.3389/fcimb.2026.1846229)
Supplement: Supplementary file 1 [file Table1.docx]

| **Supplementary table 1.** Specific Subtypes of 60 NTM patients with Eosinophilia | |
| --- | --- |
| Subtypes | N（%） |
| *Mycobacterium avium-intracellulare Complex (MAC)* | 25(41.7) |
| *Mycobacterium chelonae-abscessus Complex* | 17(28.3) |
| *M. colombiense* | 8(13.3) |
| *M. fortuitum* | 2(3.3) |
| *M. gordonae* | 2(3.3) |
| *M. chimaera* | 2(3.3) |
| *M. kansasii* | 1(1.7) |
| *M. ulcerans* | 1(1.7) |
| *M. scrofulaceum* | 1(1.7) |
| *M. szulgai* | 1(1.7) |
| Unsubtyped | 6(10.0) |
