# Supplementary material for: Clinical characteristics of peripheral blood eosinophilia in nontuberculous mycobacterial disease: a multicenter comparative retrospective study
Source: Front Cell Infect Microbiol. 2026 Aug 11;16:1846229. doi: 10.3389/fcimb.2026.1846229 (PMC13503238; doi:10.3389/fcimb.2026.1846229)
Supplement: Supplementary file 2 [file Table2.docx]

| **Supplementary table 2.** Specific Subtypes of 139 NTM patients without Eosinophilia | |
| --- | --- |
| Subtypes | N（%） |
| *Mycobacterium avium-intracellulare Complex (MAC)* | 53（38.1） |
| *Mycobacterium chelonae-abscessus Complex* | 46（33.1） |
| *M. colombiense* | 6（4.3） |
| *M. kansasii* | 5（3.6） |
| *M. paraintracellulare* | 3（2.2） |
| *M. gordonae* | 3（2.2） |
| *M. nonchromogenicum* | 2（1.4） |
| *M. mucogenicum* | 2（1.4） |
| *M. massiliense* | 2（1.4） |
| *M. shigaense* | 1（0.7） |
| *M. xenopi* | 1（0.7） |
| *M. mucilaginosum* | 1（0.7） |
| *M. smegmatis* | 1（0.7） |
| *M. triviale* | 1（0.7） |
| *M. marinum* | 1（0.7） |
| *M. madridense* | 1（0.7） |
| *M. fortuitum* | 1（0.7） |
| *M. peregrinum* | 1（0.7） |
| Unsubtyped | 16（11.5） |
