# Supplementary material for: Clinical characteristics of peripheral blood eosinophilia in nontuberculous mycobacterial disease: a multicenter comparative retrospective study
Source: Front Cell Infect Microbiol. 2026 Aug 11;16:1846229. doi: 10.3389/fcimb.2026.1846229 (PMC13503238; doi:10.3389/fcimb.2026.1846229)
Supplement: Supplementary file 3 [file Table3.docx]

| **Supplementary table 3.** Comparison of Co-infection between NTM Patients with and without Eosinophilia | | | |
| --- | --- | --- | --- |
| Variable | **Eosinophilia group**  **(n = 60)** | **Non-eosinophilia group**  **(n = 139)** | ***P*** |
| G ^+^ bacteria | 10 (16.7) | 20 (14.4) | 0.680 |
| G ^-^ bacteria | 20 (33.3) | 34 (24.5) | 0.196 |
| Virus | 11 (18.3) | 15 (10.8) | 0.147 |
| Tuberculosis | 5 (8.3) | 7 (5.0) | 0.567 |
| Fungi | 24 (40.0) | 36 (25.9) | **0.047** |
| *Talaromyces marneffei* | 12 (20.0) | 7 (5.0) | **<.001** |
| *Candidiasis* | 7 (11.7) | 16 (11.5) | 0.975 |
| *Aspergillus* | 5 (8.3) | 12 (8.6) | 0.945 |
| *Cryptococcus* | 1 (1.7) | 2 (1.4) | 1.000 |

Each patient may have more than one subtype infection.
