# Supplementary material for: Clinical characteristics of peripheral blood eosinophilia in nontuberculous mycobacterial disease: a multicenter comparative retrospective study
Source: Front Cell Infect Microbiol. 2026 Aug 11;16:1846229. doi: 10.3389/fcimb.2026.1846229 (PMC13503238; doi:10.3389/fcimb.2026.1846229)
Supplement: Supplementary file 4 [file Table4.docx]

| **Supplementary table 4.** Comparison of Clinical Characteristics Between Patients with Disseminated NTM Disease and NTM Pulmonary Disease | | | |
| --- | --- | --- | --- |
| **Characteristics** | Disseminated NTM (n=69) | NTM pulmonary disease (n=130) | *P* |
| Gender |  |  | 0.007 |
| Male | 38 (55.1%） | 46 (35.4%） |  |
| Female | 31(44.9%） | 84 (64.6%） |  |
| Age (years) | 53.0 (46.0-60.5) | 57.0 (48.0-67.3) | 0.039 |
| BMI (kg/m^2^) | 20.7 (18.3-23.2) | 19.6 (17.9-22.0) | 0.046 |
| Classification by growth rates |  |  | 0.656 |
| RGM | 25 (39.1%） | 45 (40.2%） |  |
| SGM | 38 (59.4%） | 62 (55.4%） |  |
| RGM and SGM co-infection | 1 (1.6%） | 5 (4.5%） |  |
| Underlying diseases |  |  |  |
| COPD | 2 (2.9%） | 8 (6.2%） | 0.510 |
| Bronchiectasis | 11 (15.9%） | 86 (66.2%） | <.001 |
| Malignancy | 3 (4.3%） | 8 (6.1%） | 0.829 |
| Diabetes mellitus | 5 (7.1%） | 8 (6.1%） | 1.000 |
| AIGA syndrome | 28/31 (90.3%） | 8/11 (72.7%） | 0.352 |
| **Clinical Manifestations** |  |  |  |
| Fever | 36 (52.2%） | 33 (25.3%） | <.001 |
| Night sweats | 7 (10.1%） | 12 (9.2%） | 0.835 |
| Chill | 8 (11.6%） | 2 (1.5%） | 0.006 |
| Weight loss | 23 (33.3%） | 19 (14.6%） | 0.002 |
| Cough | 45 (65.2%） | 116 (89.2%） | <.001 |
| Expectoration | 41 (59.4%） | 105 (80.8%） | 0.001 |
| Hemoptysis | 7 (10.1%） | 51 (39.2%） | <.001 |
| Chest pain | 13 (18.8%） | 18 (13.9%） | 0.355 |
| Dyspnea | 12 (17.4%） | 39 (30.0%） | 0.053 |
| Bone pain | 19 (27.5%） | 3 (2.3%） | <.001 |
| Myalgia/Arthralgia | 14 (20.3%） | 4 (3.1%） | <.001 |
| Rash/Subcutaneous nodules | 31 (44.9%） | 0 (0.0%） | <.001 |
| Superficial lymphadenopathy | 39 (56.5%） | 2 (1.5%） | <.001 |
| **Chest radiological features** |  |  |  |
| Solitary nodule/mass | 9 (18.0%) | 3 (2.3%) | <.001 |
| Multiple nodules/masses | 26 (52.0%) | 79 (60.8%) | 0.285 |
| Bronchiectasis | 11 (22.0%) | 86 (66.2%) | <.001 |
| Single cavity | 7 (14.0%) | 14 (10.8%) | 0.545 |
| Multiple cavity | 2 (4.1%) | 19 (14.7%) | 0.049 |
| Consolidation | 47 (94.0%) | 116 (89.2%) | 0.487 |
| Ground-glass opacities | 10 (20.0%) | 37 (28.5%) | 0.247 |
| Tree-in-bud sign | 5 (10.0%) | 12 (9.2%) | 1.000 |
| Emphysema/pulmonary bulla | 7 (14.0%) | 19 (14.6%) | 0.916 |
| Fibrotic changes | 40 (80.0%) | 68 (52.3%) | <.001 |
| Pleural effusion | 19 (38.0%) | 10 (7.7%) | <.001 |
| Hilar lymph node enlargement | 27 (54.0%) | 31 (23.9%) | <.001 |
| Calcifications | 11 (22.0%) | 26 (20.0%) | 0.766 |
| **Laboratory findings** |  |  |  |
| WBC (×10^9^/L) | 13.0（8.2-18.7） | 6.7（5.0-8.2） | <.001 |
| N (×10^9^/L) | 8.8（5.3-13.8） | 3.9（3.2-5.4） | <.001 |
| L (×10^9^/L) | 1.6（1.2-2.2） | 1.45（1.1-2.0） | 0.108 |
| EOS (×10^9^/L) | 0.6（0.2-0.9） | 0.1（0.1-0.3） | <.001 |
| CD4^+^T (/μL) | 735^a^（436-1047） | 541^b^（395-778） | 0.044 |
| CD8^+^T (/μL) | 504^a^（299-709） | 371^b^（229-507） | 0.017 |
| ESR（mm/h） | 73.0（39.3-91.5） | 25.0（10.0-51.0） | <.001 |
| CRP（mg/L） | 68.1（24.7-110.8） | 5.1（1.9-14.3） | <.001 |
| PCT（ng/ml） | 0.15（0.09-0.81） | 0.05（0.03-0.10） | <.001 |
| Immunoglobulin | N=48 | N=50 |  |
| IgG（g/L） | 17.3（13.8-20.8） | 12.13（10.5-14.8） | <.001 |
| IgA（g/L） | 2.8±1.4 | 2.3±0.9 | 0.028 |
| IgM（g/L） | 1.17（0.7-1.6） | 0.9（0.7-1.5） | 0.327 |
| IgE（IU/ml） | 81.3^c^（21.8-197.0） | 47.9^d^（15.9-103.6） | 0.059 |
| PLT(10^9^/L) | 369（233-478） | 235（189-298） | <.001 |
| HGB（g/L） | 100（76-119） | 121.9（110-130） | <.001 |
| ALB（g/L） | 30.8±7.9 | 38.0±5.3 | <.001 |
| GLO（g/L） | 36.6（31.1-43.3） | 31.0（26.9-34.7） | <.001 |
| BDG（pg/ml） | 10.0^e^（10.0-12.7） | 10.0^f^（10.0-19.7） | 0.941 |
| GM-test（ng/L） | 0.3^e^（0.2-0.6） | 0.3^f^（0.2-0.8） | 0.476 |
| **Co-infection** |  |  |  |
| G ^+^ bacteria | 10 (14.5%） | 20 (15.4%） | 0.867 |
| G ^-^ bacteria | 17 (24.6%） | 37 (28.5%） | 0.564 |
| Fungi | 43 (62.3%） | 27 (20.8%） | <.001 |
| *Talaromyces marneffei* | 28 (40.6%） | 0 (0.0） | <.001 |
| *Candidiasis* | 10 (14.5%） | 14 (10.8%） | 0.443 |
| *Aspergillus* | 4 (5.8%） | 13 (10.0%） | 0.313 |
| *Cryptococcus* | 2 (2.9%） | 1 (0.8%） | 0.574 |
| Virus | 15 (21.7%） | 11 (8.5%） | 0.008 |
| Tuberculosis | 6 (8.7%） | 6 (4.6%） | 0.402 |

Datae are expressed shown as the number (percentage) of patients or the median (interquartile rang) or the average±standard deviation. Data are expressed shown as the number (percentage) of patients or the median (interquartile range) or the average±standard deviation. Definition of abbreviations: BMI: body mass index; RGM: rapidly growing mycobacteria; SGM: slowly growing mycobacteria; COPD:Chronic Obstructive Pulmonary Disease; AIGA:Anti-interferon-γ autoantibody. WBC: white blood cell; N: neutrophil; L: lymphocyte; EOS: eosinophil;PLT: platelet; HGB: hemoglobin; ALB: albumin;GLO: globulin; IgG:Immunoglobulin G;IgA:Immunoglobulin A;IgM:Immunoglobulin M;IgE: Immunoglobulin E;PCT: procalcitonin； ESR: erythrocyte sedimentation rate; CRP: C-reactive protein; BDG: 1,3-β-D-glucan assay; GM test: galactomannan test.
