# Supplementary material for: Clinical characteristics of peripheral blood eosinophilia in nontuberculous mycobacterial disease: a multicenter comparative retrospective study
Source: Front Cell Infect Microbiol. 2026 Aug 11;16:1846229. doi: 10.3389/fcimb.2026.1846229 (PMC13503238; doi:10.3389/fcimb.2026.1846229)
Supplement: Supplementary file 6 [file Table6.docx]

| **Supplementary Table 6.** Sensitivity analysis EOS continuous | | | |
| --- | --- | --- | --- |
| **Characteristic** | OR | 95%CI | *P* |
| Eosinophilia | 4.948 | 1.492-7.589 | **< 0.001** |
| Age | 0.992 | 0.962-1.019 | 0.593 |
| Female | 1.029 | 0.486-2.353 | 0.952 |
| Bronchiectasis | 0.211 | 0.06-0.335 | **< 0.001** |
| COPD | 2.924 | 0.021-4.169 | 0.542 |
| CTD | 3.360 | 0.55-23.913 | 0.199 |
| Virus co-infection | 2.116 | 0.724-6.305 | 0.171 |
| Fungi co-infection | 3.669 | 2.349-11.111 | **0.002** |
| Definition of abbreviations: COPD: Chronic Obstructive Pulmonary Disease; CTD: Connective Tissue Diseases. | | | |
