# Supplementary material for: Clinical characteristics of peripheral blood eosinophilia in nontuberculous mycobacterial disease: a multicenter comparative retrospective study
Source: Front Cell Infect Microbiol. 2026 Aug 11;16:1846229. doi: 10.3389/fcimb.2026.1846229 (PMC13503238; doi:10.3389/fcimb.2026.1846229)
Supplement: Supplementary file 7 [file Table7.docx]

| **Supplementary Table 7.** Sensitivity analysis LASSO | | | |
| --- | --- | --- | --- |
| **Characteristic** | OR | 95%CI | *P* |
| Eosinophilia | 4.693 | 2.053-11.044 | **< 0.001** |
| hypertension | 0.143 | 0.029-0.571 | **0.004** |
| hyperlipemia | 0.199 | 0.001-1.904 | 0.191 |
| Bronchiectasis | 0.160 | 0.065-0.366 | **< 0.001** |
| Chronic rhinitis and pharyngitis | 0.229 | 0.002-2.550 | 0.272 |
| CTD | 3.488 | 0.529-29.575 | 0.199 |
| Virus co-infection | 3.109 | 1.011-10.171 | **0.048** |
| Fungi co-infection | 4.449 | 2.027-10.122 | **< 0.001** |
| Definition of abbreviations: COPD: Chronic Obstructive Pulmonary Disease; CTD: Connective Tissue Diseases. | | | |
